# Early Detection of Elevated Ketone Bodies in Type 1 Diabetes Using Insulin and Glucose Dynamics Across Age Groups: Model Development Study

**DOI:** 10.2196/67867

**Published:** 2025-04-10

**Authors:** Simon Cichosz, Clara Bender

**Affiliations:** 1 Department of Health Science and Technology, Aalborg University, Selma Lagerløfs Vej 249, Aalborg, 9260, Denmark, 45 99403809

**Keywords:** type 1 diabetes, machine learning, diabetic ketoacidosis, ketone level, diabetic complication, prediction model

## Abstract

**Background:**

Diabetic ketoacidosis represents a significant and potentially life-threatening complication of diabetes, predominantly observed in individuals with type 1 diabetes (T1D). Studies have documented suboptimal adherence to diabetes management among children and adolescents, as evidenced by deficient ketone monitoring practices.

**Objective:**

The aim of the study was to explore the potential for prediction of elevated ketone bodies from continuous glucose monitoring (CGM) and insulin data in pediatric and adult patients with T1D using a closed-loop system.

**Methods:**

Participants used the Dexcom G6 CGM system and the iLet Bionic Pancreas system for insulin administration for up to 13 weeks. We used supervised binary classification machine learning, incorporating feature engineering to identify elevated ketone bodies (>0.6 mmol/L). Features were derived from CGM, insulin delivery data, and self-monitoring of blood glucose to develop an extreme gradient boosting-based prediction model. A total of 259 participants aged 6-79 years with over 49,000 days of full-time monitoring were included in the study.

**Results:**

Among the participants, 1768 ketone samples were eligible for modeling, including 383 event samples with elevated ketone bodies (≥0.6 mmol/L). Insulin, self-monitoring of blood glucose, and current glucose measurements provided discriminative information on elevated ketone bodies (receiver operating characteristic area under the curve [ROC-AUC] 0.64‐0.69). The CGM-derived features exhibited stronger discrimination (ROC-AUC 0.75‐0.76). Integration of all feature types resulted in an ROC-AUC estimate of 0.82 (SD 0.01) and a precision recall-AUC of 0.53 (SD 0.03).

**Conclusions:**

CGM and insulin data present a valuable avenue for early prediction of patients at risk of elevated ketone bodies. Furthermore, our findings indicate the potential application of such predictive models in both pediatric and adult populations with T1D.

## Introduction

Diabetic ketoacidosis (DKA) represents a significant and potentially life-threatening complication of diabetes, predominantly observed in individuals with type 1 diabetes (T1D), although occurrences in those with type 2 diabetes are not uncommon [1,2]. DKA arises from an inadequate supply of insulin, leading to dysregulation of blood glucose levels. Consequently, the body resorts to metabolizing fat for energy, resulting in the accumulation of ketone bodies in the bloodstream alongside elevated blood sugar levels. This metabolic disturbance manifests in symptoms such as nausea, vomiting, abdominal pain, confusion, excessive thirst, and frequent urination [1]. If left untreated, DKA can progress to coma and, in severe cases to mortality, necessitating immediate medical intervention comprising insulin administration and fluid replacement to restore normal blood glucose and ketone levels [3] .

Children and adolescents are particularly susceptible to DKA due to their ongoing growth and development, which introduce complexities in diabetes management [4]. Factors such as missed insulin doses, illness, or infection can rapidly precipitate DKA in this demographic.

Studies have documented suboptimal adherence to diabetes management among children and adolescents, as evidenced by deficient ketone monitoring practices [5-7] . For instance, a recent study involving 2995 participants revealed that a significant proportion lacked ketone testing supplies at home, with a considerable proportion reporting infrequent ketone checks, particularly in instances of elevated glucose levels [7].

Closed-loop systems offer a promising approach to addressing the challenges of diabetes management in both pediatric and adult populations [8,9]. Leveraging CGM technology provides real-time feedback on blood glucose levels, facilitating automated adjustments to insulin delivery via an insulin pump. By delivering precise insulin doses tailored to individual glucose fluctuations, closed-loop systems can reduce the risks of both hypoglycemia and hyperglycemia, thereby diminishing the likelihood of DKA development. However, this technology does not eliminate the risk of DKA [10-12].

A recent study by Cichosz and Bender [13] demonstrated the potential of CGM data in predicting elevated ketone levels among adults with T1D. However, such investigations remain scarce in pediatric populations and have not incorporated insulin data. Consequently, this study aims to explore the predictive potential of CGM and insulin data for elevated ketone bodies in pediatric and adult patients with T1D using a closed-loop system.

## Methods

### Data Sources

To ascertain whether patterns derived from CGM and insulin usage could serve as predictive indicators for elevated ketone bodies—a potential risk factor for DKA in individuals with diabetes—data sourced from the intervention arm of The Insulin-Only Bionic Pancreas Pivotal Trial (NCT04200313) [14] were analyzed. This trial constituted a multicenter randomized controlled study comparing an at-home closed-loop system with the prevailing standard of care.

The participant cohort encompassed individuals diagnosed with T1D aged 6 to 79 years. Participants used the Dexcom G6 CGM system in conjunction with the iLet Bionic Pancreas system for insulin administration for up to 13 weeks. Additionally, participants were equipped with a blood ketone meter and test strips and were provided instructions to measure ketone levels if glucose readings surpassed 300 mg/dL. The intervention group comprised 219 patients with T1D, exhibiting a mean glycated hemoglobin of 7.9 (SD 1.2%); 63 mmol/mol with a mean age of 28 (SD 19) years, and a female representation of 49% (n=107) within the cohort.

For this analysis, inclusion criteria required the presence of ketone measurements along with corresponding CGM and insulin data within a 12-hour timeframe preceding the ketone measurements. CGM data periods had to demonstrate a wear time of ≥50% to be considered for inclusion. Given the sampling rate of the CGM system of 12 readings per hour, inclusion mandated a minimum of 72 glucose samples within the 12-hour observation window.

This study adheres to the recommended guidelines delineated in the “Transparent Reporting of a Multivariable Prediction Model for Individual Prognosis or Diagnosis” (TRIPOD).

### Model Target

In this study, we used a supervised binary classification machine learning methodology to discern elevated ketone bodies. It is well established that ketone levels below 0.6 mmol/L fall within the reference range, whereas levels at or above 0.6 mmol/L pose a significantly augmented risk of DKA [15]. Therefore, we defined the binary classification task as the identification of elevated ketone bodies (≥0.6 mmol/L) versus nonelevated ketone bodies (<0.6 mmol/L) during episodes of elevated glucose readings.

### Feature Engineering

Feature engineering is a process within machine learning wherein new features are generated from raw data through a series of transformations, aggregations, or extractions of information from existing variables. The primary objective is to enhance the performance of machine learning algorithms by constructing new features that more accurately capture the underlying relationships in the data, thereby augmenting prediction accuracy and model effectiveness [16]. To identify the most relevant predictors of elevated ketone levels in patients with diabetes, we explored a broad range of potential features over the preceding 12-hour period. This included absolute values, summations, and dynamic patterns to capture temporal variations. Given the limited literature on the most effective individual features or their optimal combinations for detecting ketone elevation, our approach aimed to systematically identify the best subset of predictors.

**Figure 1.**
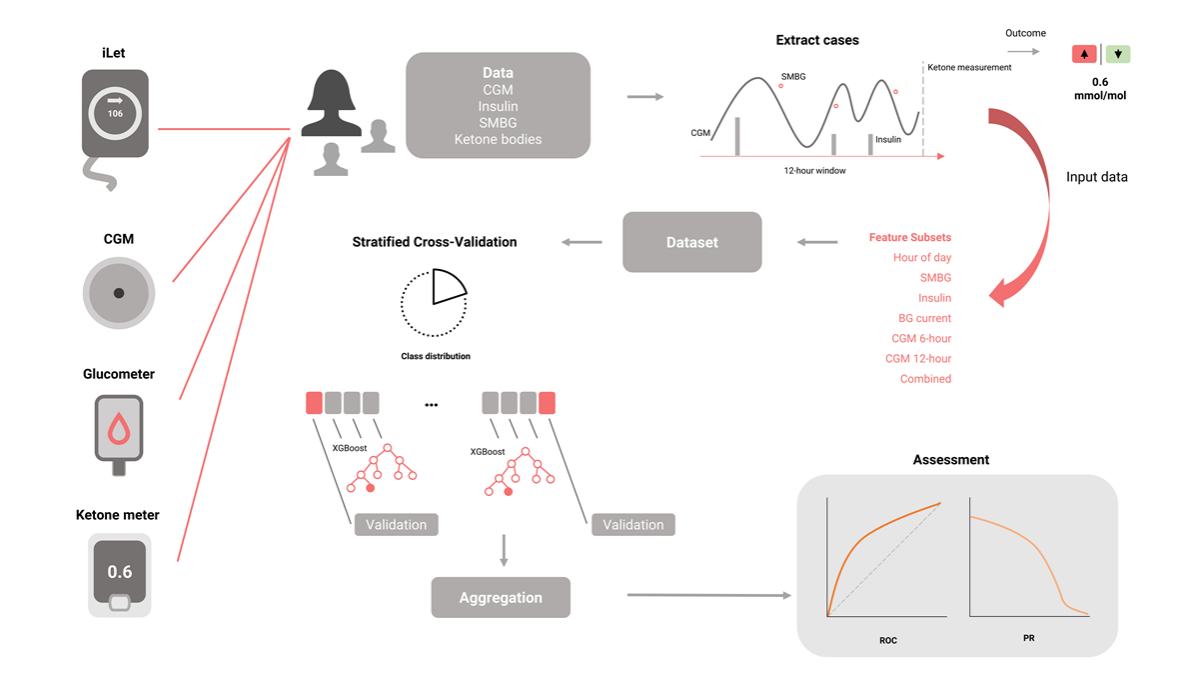
Overview of the data pipeline for predicting ketone levels using machine learning. Data from multiple sources, including the iLet closed-loop system, continuous glucose monitor (CGM), glucometer, and ketone meter, are collected and processed. A window of CGM, insulin, and self-monitored blood glucose (SMBG) data is extracted for feature engineering. Various feature subsets, such as the hour of day, SMBG, insulin, and CGM trends over different timeframes, are used as input to train a model. Stratified cross-validation ensures balanced class distribution, and model performance is evaluated using receiver operating characteristic (ROC) and precision-recall (PR) curves.

A total of 26 features were extracted from CGM, insulin data, and glucose meter readings within a 6- and 12-hour window preceding the ketone samples, as depicted in Figure 1. Table 1 enumerates the features extracted from each data source. These features encompassed mathematical transformations of the signals to characterize their dynamics, range of variation, cumulative effects, distribution, and extreme values. The methodology adopted was data-driven and exploratory, devoid of prior assumptions regarding which features of the signal that would yield optimal discriminative information when combined. The dynamics of glucose levels are intricately shaped by diurnal patterns, influenced by factors such as dietary intake, basal and bolus insulin administration, endocrine activity, and behavioral habits including physical exertion and sleep.

**Table 1.** Extracted features for CGM^a^, insulin, and BGM^b^ data. Features marked with “√” indicate inclusion for the respective data type and time division.

Feature	CGM (6h)	CGM (12h)	Insulin basal (6‐12h)^c^	Insulin bolus (6‐12h)^c^	Insulin meal (6‐12h)^c^	BGM (12h)
Latest	√	√				
Maximum	√	√				√
Minimum	√	√				√
Sum			√	√	√	
Mean	√	√				
Standard deviation	√	√				
Time spent when blood glucose levels >300 mg/dL	√	√				
Decreases ratio	√	√				
Mean decrease	√	√				
Hour of the day	√					

a CGM: continuous glucose monitoring.

b BGM: blood glucose monitoring.

c Insulin features are extracted from both a 0‐6 hour and a 6‐12 hour window.

While many features entailed straightforward mathematical derivatives such as summations, standard deviations, and the proportion of time spent above 300 mg/dL blood glucose levels, we additionally incorporated a metric assessing the rate of glucose decline relative to preceding measurements to capture finer-scale dynamics within the glucose signal. The formulation for this calculation is delineated below:


$$cgm=[x_{1},x_{2},x_{3}...x_{n}]$$

$$\begin{array}{l} Decreases_{n}=\sum_{i=1}^{n-1}\left\{ \begin{array}{l} 1^{if_{i+1}-x_{i}§lt;0}\\ 0_{otherwise} \end{array}\right. \end{array}$$

$$Decrease_{ratio}=\frac{decreases_{n}}{/cgm/}$$

### Model Development

For model development, we used a supervised binary classification approach using an extreme gradient boosting (XGBoost) classifier to predict elevated levels of ketone bodies. XGBoost is a renowned machine learning algorithm known for its ability to handle intricate datasets by amalgamating weak prediction models (decision trees) into a robust ensemble [17]. It excels in capturing nonlinear relationships, managing missing or imbalanced data, and mitigating overfitting, thereby typically yielding high predictive performance. This efficacy has been demonstrated in clinical prediction models across a spectrum of medical domains [18-21].

The model was trained using features from each data type individually and in combination, aiming to ascertain their predictive capacity for the target variable. We used 5-fold stratified cross-validation to ensure an unbiased estimation of the model’s performance and hyperparameter estimation, with stratification ensuring uniform proportions of events across folds [22]. The following parameters were optimized using a grid search strategy: learning rate (0.01, 0.1, 0.3), number of estimators (50, 100, 150), max depth (2, 4, 8), minimum child weight (1, 3, 5), subsample (0.6, 0.8, 1.0), and γ (0, 1, 5).

All analyses were conducted using MATLAB (version R2021b; MathWorks) and Python (version 3; Python Software Foundation), leveraging the Scikit-learn package (version 0.23.2) for machine learning utilities, the SHapley Additive exPlanations (SHAP) package (version 0.43.0) for interpretability assessment, and the XGBoost package (version 1.7.5) for implementing the classifier.

### Model Assessment and Interpretability

The discriminative performance of the model was assessed using the computation of the area under the receiver operating characteristic curve (ROC-AUC) and the area under the precision-recall curve (PR-AUC) [23]. The uncertainty of estimates was calculated as the SD across folds. To enhance model interpretability, SHAP average values across folds were leveraged for explanatory purposes. These values offer insights into the contribution of individual features towards model predictions, thereby enhancing the interpretability and transparency of the modeling process [24]. .

### Sensitivity Analysis

In a sensitivity analysis, we restricted the subgroup to patients aged <18 years. The objective of this analysis was to assess the model’s performance in pediatric and adolescent patients, as these groups have a higher risk of developing DKA [25]. The objective was to test whether any substantial difference was observed in ROC-AUC performance in patients under 18 years.

### Ethical Considerations

This study is a reanalysis of existing and anonymized data from the Insulin Only Bionic Pancreas Pivotal Trial [14]. According to Danish law (Komitéloven, kap. 4, § 14, stk. 3) on the ethical review of health science research projects and health data science research projects, this study did not require approval from an institutional or licensing committee.

The original Insulin Only Bionic Pancreas Pivotal Trial protocol and informed consent forms were approved by institutional review boards. Written informed consent was obtained from each participant prior to enrollment. An independent data and safety monitoring board provided trial oversight reviewing unmasked safety data during the conduct of the study.

We confirm that all methods were carried out in accordance with relevant guidelines and regulations. The data was accessed and analyzed in an anonymized form.

## Results

### Participant Characteristics

In total, 259 patients (n=93 for patients aged <18 years) were included in the analysis. Another 181 patients did not have qualified ketone measurements with a CGM window (n=71) or were part of the control group, which did not use a connected insulin pump (n=110). Among the included patients, 1768 ketone samples were eligible for modeling, including 383 event samples with ketone levels ≥0.6 mmol/L. Overall, the patients had over 14,300,000 CGM measurements, corresponding to over 49,000 days of full-time monitoring.

### Model Performance

**Figure 2.**
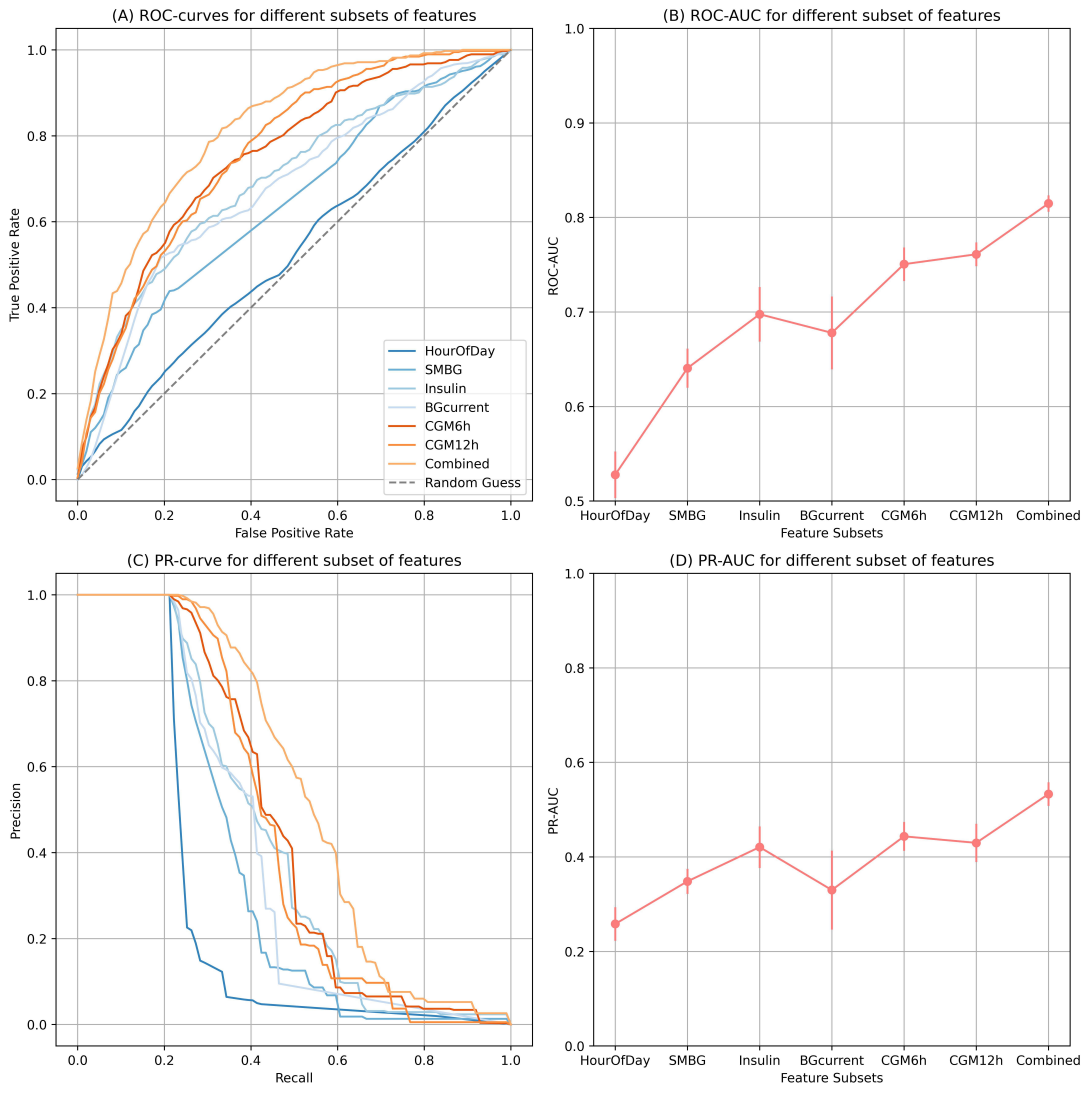
For endpoint 1, (A) ROC-curves for different subsets of features, (B) ROC-AUC for different subset of features , (C) PR-curve for different subset of features, (D) PR-AUC for different subset of features. AUC: area under the curve; BG: blood glucose; CGM: continuous glucose monitoring; PR: precision recall; ROC: receiver operating characteristics; SMBG: self-monitoring of blood glucose.

The ROC-AUC, PR-AUC, and individual curves are presented in Figure 2. The plots illustrate the performance of adding individual datatypes and a combined estimate. Insulin, self-monitoring of blood glucose (SMBG), and current glucose measurements, all provided discriminative information on elevated ketone bodies (ROC-AUC 0.64‐0.69). The features derived from the CGM window demonstrated greater discrimination (ROC-AUC 0.75‐0.76). Notably, extending the CGM window from6 hours to 12 hours only added minimal discriminative power, as measured by ROC-AUC. Combining all feature types yielded an ROC-AUC estimate of 0.82 (SD 0.01) and a PR-AUC of 0.53 (SD 0.03). In the sensitivity analysis including only pediatric patients (age <18 years), the ROC-AUC estimate was 0.80 (SD 0.01). The final selected hyperparameters were a learning rate of 0.2, 100 estimators, a maximum depth of 8, a minimum child weight of 1, a subsample ratio of 1.0, and a γ value of 1.

### Interpretability

**Figure 3.**
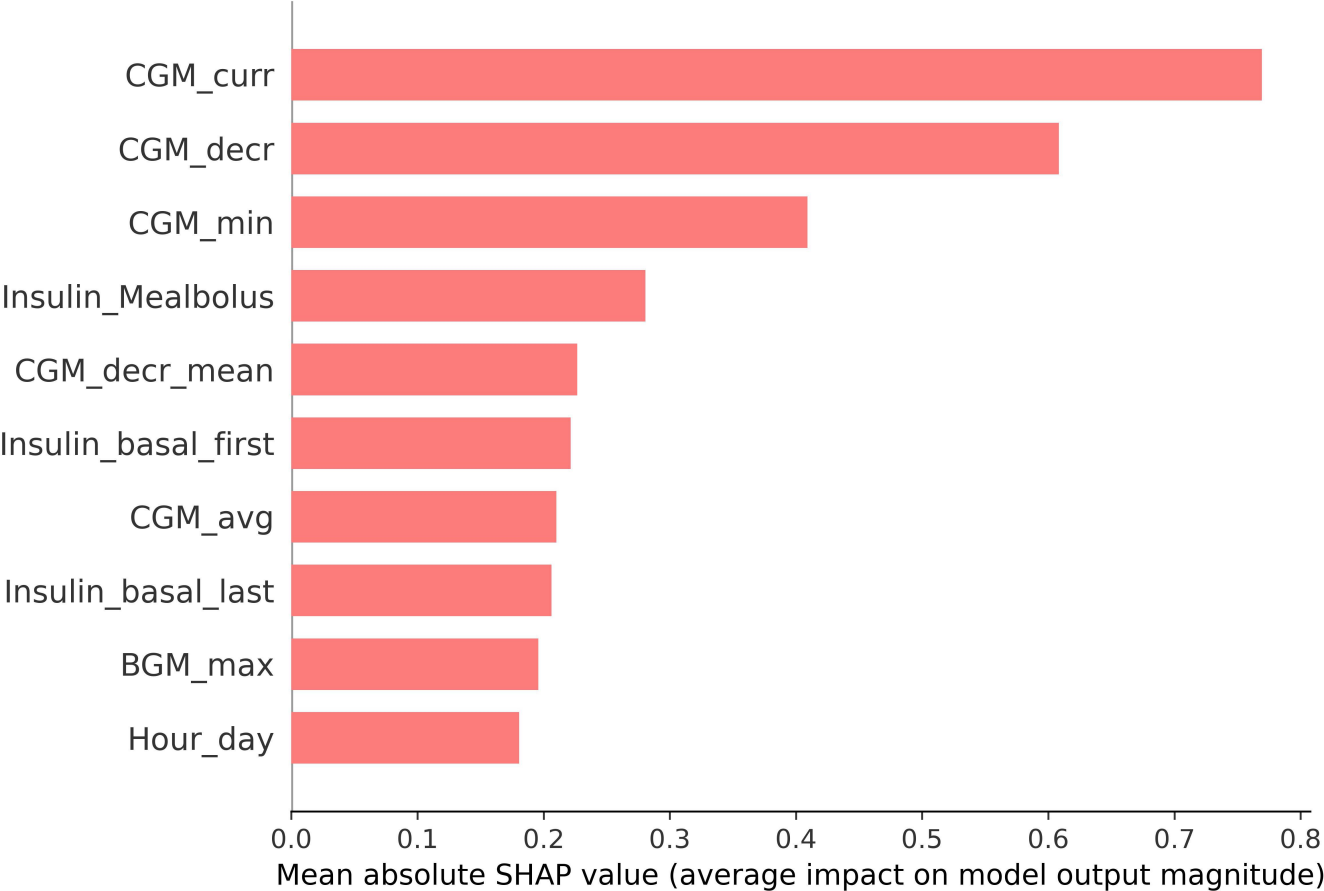
SHAP bar plot illustrating the 10 features with most important features in the model’s prediction. BGM: blood glucose measurement; CGM: continuous glucose monitoring; Avg: average; Min: minimum; Decr: decrease; Max: maximum.

Feature importance analysis including the combined features showed that data from both CGM and insulin deliveries adds significant information to the models’ predictive capabilities. The mean SHAP values for the 10 highest-ranking features are presented in Figure 3. Furthermore, a SHAP Beeswarm plot is provided in Multimedia Appendix 1. As expected, the current CGM value had the highest contribution, followed by the ratio of decrease in the CGM window. Further, insulin-related features such as meal bolus and basal insulin deliveries had significant impacts.

### Patient Example

**Figure 4.**
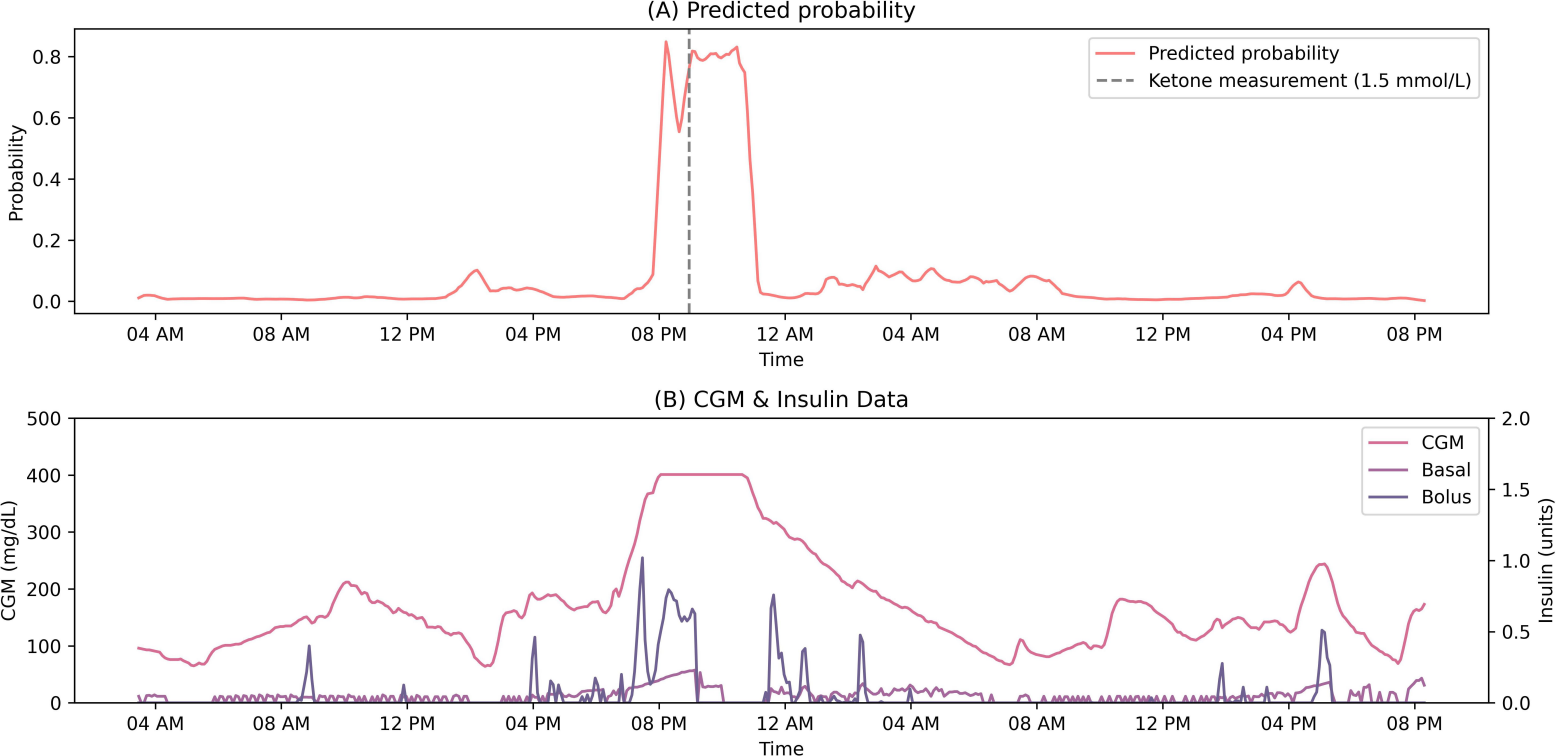
Patient example: (A) Predicted probability for elevated ketone bodies over two days of monitoring; (B) The corresponding CGM and insulin inputs to the model. CGM: continuous glucose monitoring.

An illustrative depiction of the predicted probability, representing the model output for elevated ketone bodies, is presented alongside CGM data and insulin delivery records for a specific patient in Figure 4. Notably, the probability of a heightened risk of elevated ketone bodies increases around 8 PM, coinciding with a ketone meter measurement confirming elevated ketones at 9 PM. This example underscores the potential utility of a predictive model, such as the one proposed in our study, for identifying impending instances of elevated ketone levels based on continuous monitoring of patients’ data. Such a model holds promise for alerting patients to take timely action, thereby mitigating the progression of adverse developments associated with diabetic ketoacidosis.

## Discussion

### Principal Findings

The objective of this investigation was to formulate and assess various data sources, including CGM, insulin, and SMBG, as potential inputs for a prediction model designed to provide timely alerts regarding the risk of developing DKA through elevated ketone body levels. The findings underscore the potential utility of patterns derived from CGM data obtained from individuals with T1D in identifying and signaling patients at risk of elevated ketone levels. It is imperative to note that elevated ketone levels serve as a precursor to DKA, a critical and potentially life-threatening complication of diabetes.

We previously showed that CGM data could be used for prediction of elevated ketone bodies in an adult population with T1D [13]. The present findings validate this observation and expand on the initial findings by examining the added predictive value of insulin and SMBG data. Furthermore, this study strongly indicates that this approach is applicable to both pediatric and adult individuals. To our knowledge, this study, along with our previously published study, is the first to explore the potential of predicting elevated ketone bodies using a combination of CGM and insulin data. However, numerous studies have reported the usage of CGM for prediction of other complications related to diabetes and diabetes treatment, such as hypoglycemia, gastroparesis, and future glucose levels [26-31].

The clinical implications of implementing a system based on the proposed model in our study are vividly illustrated through the patient’s continuous data depicted in Figure 4. The predicted risk or probability of elevated ketone bodies offers patients a more nuanced and informative warning compared to solely relying on glucose levels. This enhanced information could prompt early intervention to prevent further progression to DKA. Potential actions triggered by these alerts may include promptly checking ketone bodies using a ketone meter, verifying the functionality of the infusion set to ensure proper insulin delivery, and corroborating CGM measurements with SMBG readings. By facilitating proactive measures, such a system has the potential to significantly mitigate the risk of adverse outcomes associated with DKA.

### Limitations

Despite the robust design of our study, which encompassed a substantial dataset and measures to estimate generalizability, several limitations warrant acknowledgment. First, while our analysis involved a sample size of 259 individuals with numerous measurements of ketone bodies (n=1768), the number of outcome events (elevated ketone levels ≥0.6 mmol/L) remained relatively small (n=383). This limited number of outcome events is reflected in the SD of the estimate observed in the ROC-AUC. Consequently, the reliability of our model’s performance on new data remains uncertain, despite indicative evidence of valuable information within the dataset. These findings need to be validated in independent datasets. An avenue for potential improvement lies in the exploration of larger datasets to enhance predictive performance and further validate these findings. While our study encompassed a diverse population spanning children, adolescents, and adults, the analysis did not delve into subgroup-specific performance. Consequently, the efficacy of our predictive model across distinct subgroups remains unexplored, potentially subject to interindividual variability. Future investigations could address this limitation by conducting subgroup analyses to elucidate performance variations across demographic or clinical strata. Our findings from patients using closed-loop insulin delivery technology cannot be extrapolated to other treatment regimens without further investigation. A key limitation is that participants only measured ketones during prolonged hyperglycemia, which, coupled with generally low adherence and possible medication influences (eg, sodium-glucose cotransporter-2 inhibitors), may introduce selection bias. Importantly, ketone levels serve as surrogate outcomes and do not necessarily predict ketoacidosis events.

### Conclusion

The innovative methodology used in this study for detecting elevated ketone levels among individuals with t1D underscores the potential of integrating CGM and insulin data as a valuable resource for early prediction of patients at risk. Moreover, our findings suggest that such a predictive model holds promise for application in both pediatric and adult populations with T1D, particularly within closed-loop systems.

Future studies are imperative to validate the robustness and reliability of these findings. Furthermore, there is a need for comprehensive investigations to assess the real-world impact of implementing a system based on the proposed prediction model. Such investigations will be instrumental in elucidating the efficacy and practical implications of leveraging predictive modeling in clinical practice for proactive management of diabetes-related complications, including DKA.

## Supplementary material

10.2196/67867 Multimedia Appendix 1 SHAP Beeswarm plot

## Abbreviations

AUC: area under the curve
CGM: continuous glucose monitoring
DKA: diabetic ketoacidosis
PR: precision recall
ROC: receiver operating characteristic
SHAP: SHapley additive exPlanations
T1D: type 1 diabetes
TRIPOD: Transparent Reporting of a Multivariable Prediction Model for Individual Prognosis or Diagnosis
XGBoost: extreme gradient boosting
