# Supplementary material for: Early Detection of Elevated Ketone Bodies in Type 1 Diabetes Using Insulin and Glucose Dynamics Across Age Groups: Model Development Study
Source: JMIR Diabetes. 2025 Apr 10;10:e67867. doi: 10.2196/67867 (PMC12005466; doi:10.2196/67867)
Supplement: Multimedia Appendix 1 [file diabetes-v10-e67867-s001.docx]

| Supplementary material |
| --- |

**SHAP Beeswarm plot**


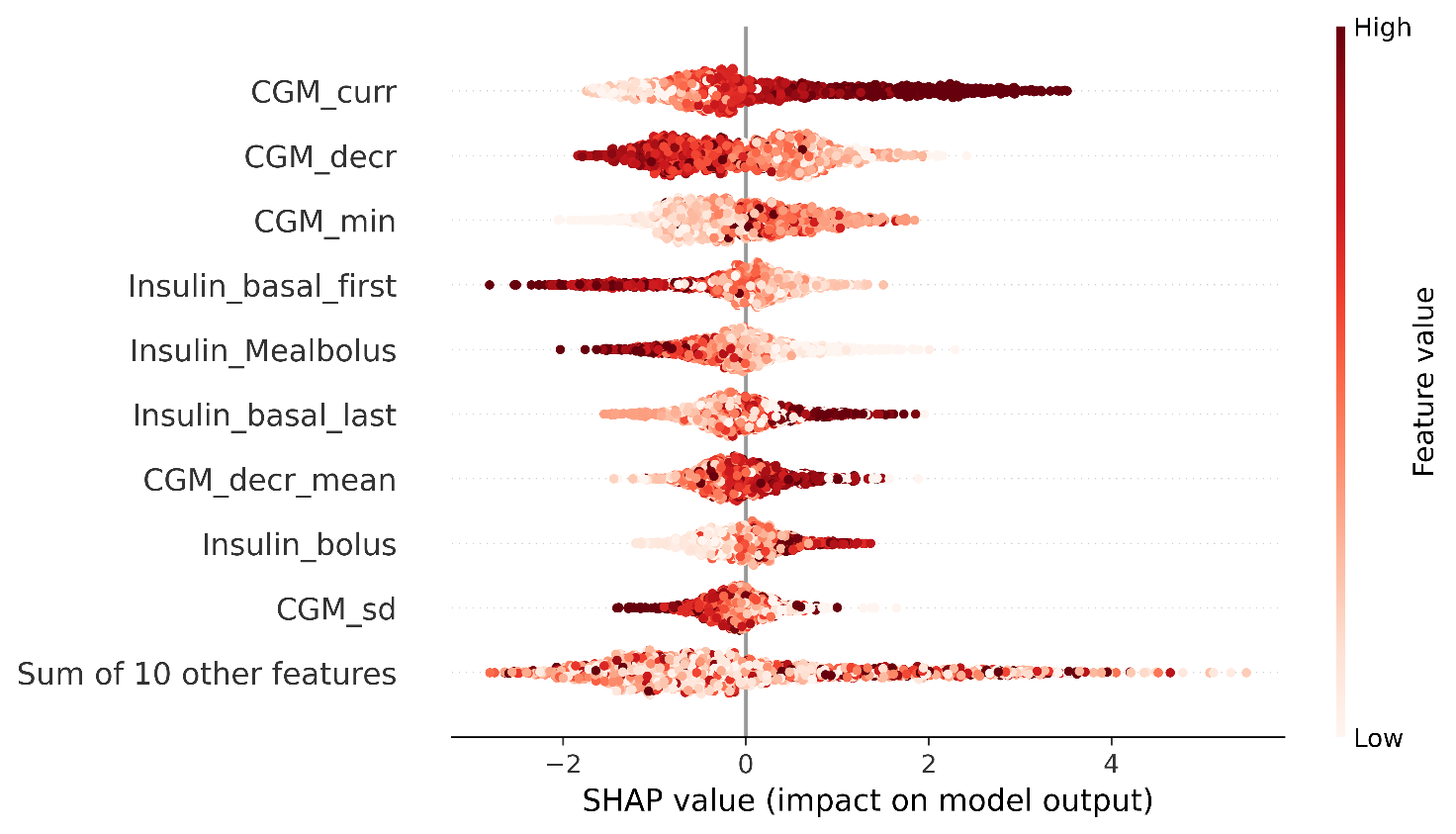


*Figure s1 - This beeswarm plot visualizes SHAP values, representing the impact of individual features on the model's predictions. Each dot corresponds to a single instance in the dataset*
